# A randomised controlled intervention study investigating the efficacy of carotenoid-rich fruits and vegetables and extra-virgin olive oil on attenuating sarcopenic symptomology in overweight and obese older adults during energy intake restriction: protocol paper

**DOI:** 10.1186/s12877-017-0700-4

**Published:** 2018-01-05

**Authors:** Anthony Villani, Hattie Wright, Gary Slater, Jonathan Buckley

**Affiliations:** 1 School of Health and Sport Sciences, University of the Sunshine Coast, Sippy Downs, QLD Australia; 2 Alliance for Research in Exercise, Nutrition and Activity (ARENA), Sansom Institute for Health Research, University of South Australia, Adelaide, Australia

**Keywords:** Obesity, Older adults, Weight loss, Carotenoids, Polyphenols, Extra-virgin olive oil, Fruit and vegetables

## Abstract

**Background:**

Weight loss interventions have not been advocated for overweight/obese older adults due to potential loss of skeletal muscle and strength impacting on physical function with potential loss of independence. Carotenoids and polyphenols are inversely associated with sarcopenic symptomology. This paper reports the protocol of a study evaluating the efficacy of a high-protein, energy restricted diet rich in carotenoids and polyphenols on body composition, muscle strength, physical performance and quality of life in overweight and obese older adults.

**Methods:**

This randomised controlled clinical trial will recruit community-dwelling, healthy overweight and obese older adults (≥60 years) for a 12-week weight loss intervention. Seventy-three participants will be recruited and randomized to an energy restricted (~30% restriction), isocaloric diet (30% protein; 30% carbohydrate; 40% fat) enriched with either: a) 375 g/d of high carotenoid vegetables, 300 g/d high carotenoid fruit, and 40-60 ml extra-virgin olive oil (EVOO); or b) 375 g/d of lower carotenoid vegetables, 300 g/d lower carotenoid fruit, and 40-60 ml Polyunsaturated fatty acid (PUFA) based oil. All participants will receive individual dietary counselling each fortnight for the duration of the study and will be asked to maintain their habitual level of physical activity throughout the study. The primary outcome will be appendicular skeletal muscle (ASM) assessed by dual energy X-ray absorptiometry (DXA). Secondary outcomes will include body weight, fat-free mass (FFM), fat mass (FM), muscle strength (Isometric hand-grip strength), physical performance (Short Physical Performance Battery), physical activity (International Physical Activity Questionnaire) and health related quality of life (SF-36). Outcomes will be measured at baseline and at week 12.

**Discussion:**

The results of this study will provide a novel insight relating to the potential influence of high carotenoid and polyphenol intakes on attenuation of ASM during dietary energy-restricted weight loss in overweight and obese older adults.

**Trial registration:**

The trial was registered on the Australia New Zealand Clinical Trials Register (ACTRN12616001400459); Trial registration date: 10th October, 2016.

## Background

Global trends in population ageing are pronounced and historically unprecedented leading to an increased need to promote healthy ageing. It is estimated that approximately one-quarter of the population in the United Kingdom (UK), Australia and in the United States (US) will be aged 65 years and older by 2050 [1, 2]. A major concern for an ageing population is potential loss of independence, institutionalisation and disability due to an age-related decline in skeletal muscle mass (SMM) and function and/or sarcopenia [3, 4].

Ageing is associated with changes in body composition that typically results in a reduction in muscle size and quality, and concomitant accumulation of fat mass (FM) [5]. The coexistence of diminished muscle mass coupled with reduced physical performance such as gait speed and/or muscle strength in addition with excessive adiposity has been termed ‘sarcopenic obesity’ [6]; this has important functional implications for older adults as there is a growing body of literature suggesting that sarcopenic obesity exacerbates age related decline in physical function which promotes frailty, physical inactivity, impairs quality of life and loss of independence [7, 8]. As a result of the rising prevalence of obesity in older adults, there are suggestions that the most common phenotype of frailty in the future may be an obese, disabled older person [9]. Therefore, effective interventions are required to facilitate improved health outcomes and enhance the quality of life of overweight and obese older adults.

The presentation of overweight and obesity in older adults presents a complex challenge to healthcare professionals for the prescription of appropriate interventions. In particular, a conundrum is the negative consequences of energy restricted diets on the loss of SMM, bone mineral content and muscle strength [10]. On average, approximately 25% of body mass lost by dietary energy restriction consists of fat-free mass (FFM) [10–12]. Due to the potential exacerbation of sarcopenic symptomology and functional decline, weight loss has not traditionally been recommended for obese older adults [13]. The addition of resistance training to an energy restricted diet has previously been shown to attenuate the loss of skeletal muscle, increase muscle strength and ameliorate frailty in obese older adults relative to either intervention alone [14–16]. However, a subset of obese older adults are limited in their capacity to engage in physical activity due to excess adiposity, reduced muscle strength and functional impairment [17]. Moreover, individuals with functional disability are unlikely to participate in physical activity at an intensity sufficient to induce a negative energy balance or to fully preserve lean tissue mass [18]. Therefore, novel dietary strategies to preserve SMM and strength during energy intake restriction are warranted.

Manipulating dietary composition during energy intake restriction, in particular protein intake, may attenuate the loss of SMM and strength. To date, most geriatric obesity weight loss trials have adopted dietary strategies involving the manipulation of dietary protein to preserve lean tissue during energy intake restriction [19–26]. Dietary protein intake stimulates muscle protein synthesis (MPS) and facilitates postprandial muscle protein accretion [27, 28]. Despite earlier studies suggesting that a decline in basal MPS may contribute to skeletal muscle wasting in the elderly [29, 30]; it appears that the elderly may be less able to efficiently utilize amino acids for MPS, resulting in a blunted muscle protein synthetic response to an anabolic stimuli, a phenomenon termed anabolic resistance [27, 31]. As of consequence, evidence based recommendations for protein intake in older adults have recently been published [32, 33]. However, there are no specific recommendations for obese older adults during energy intake restriction. Weijs and Wolfe [20] recently showed that at least 1.2 g/kg body weight or 1.9 g/kg FFM as optimal daily protein intake for obese older adults during energy intake restriction. Despite this however, an important determinant of MPS is not only an adequate intake of dietary protein. Plasma levels of antioxidants, in particular carotenoids and polyphenols, have shown to be inversely associated with sarcopenic symptomology and frailty [34–38]. Similar observations have been observed for fruit and vegetable intake [39–42]. Furthermore, a number of observational studies have reported that greater adherence to a Mediterranean Diet, characterized by a high intake of carotenoids from fruits and vegetables and polyphenols from extra-virgin olive oil (EVOO), is associated with lower odds of sarcopenia, frailty and improved mobility and physical performance [43–51]. Vegetable intake, in particular green-leafy vegetables, has shown to be associated with increased levels of physical activity [52]. In addition, increases in plasma lutein through supplementation are correlated with increases in physical activity and reductions in sedentary time in older adults [53]. Thus, an increase in carotenoids may attenuate loss of SMM by increasing physical activity during dietary energy restriction. The efficacy of carotenoid rich fruits and vegetables plus EVOO when coupled with a high protein, energy restricted diet however is unknown.

The aim of this study is to investigate the effect of a high protein diet rich in carotenoids from fruits and vegetables and polyphenols from EVOO on appendicular skeletal muscle (ASM) in overweight and obese older adults during dietary energy-restricted weight loss. Secondary outcomes included: body weight, FFM, fat mass (FM), muscle strength, physical performance and health related quality of life. The current paper presents the protocol for the recruitment and randomisation, study design, dietary intervention and key outcome assessments.

## Methods

### Study design, participants and recruitment

This is intended to be a 12-week randomised controlled trial (RCT) involving overweight and obese older adults aged ≥60 years. Community dwelling healthy older adults will be recruited from the Sunshine Coast region, Queensland, Australia via local flyers, newspaper advertisements and social media. Participants who express interest will be screened over the telephone by research personnel against eligibility criteria, outlined in Table 1. Body mass index (BMI) is one of the more common methods used to assess obesity in primary care and in subspecialty settings. Considering the recent evidence related to all-cause mortality risk and BMI in older adults [54], participants will be eligible to participate in the study based on a BMI classification of ≥27 kg/m^2^, consistent with previous geriatric obesity reduction trials [19–23].

**Table 1:** Participant eligibility criteria

Inclusion Criteria	Exclusion Criteria
Aged ≥60 years	Poor cognition/unable to follow study protocol/unable to provide informed consent
Community-dwelling	Type 2 diabetes mellitus
Overweight or obese (BMI: ≥27 kg/m^2^)	Liver disease, unstable cardiovascular disease, respiratory, neurological or gastrointestinal diseases
	Malignancy
	Current or recent use of anti-inflammatory drugs, corticosteroid agents or sex steroid compounds
	No current involvement in a secondary study at the time of enrolment

*Abbreviations: BMI* Body Mass Index

**Fig. 1:**
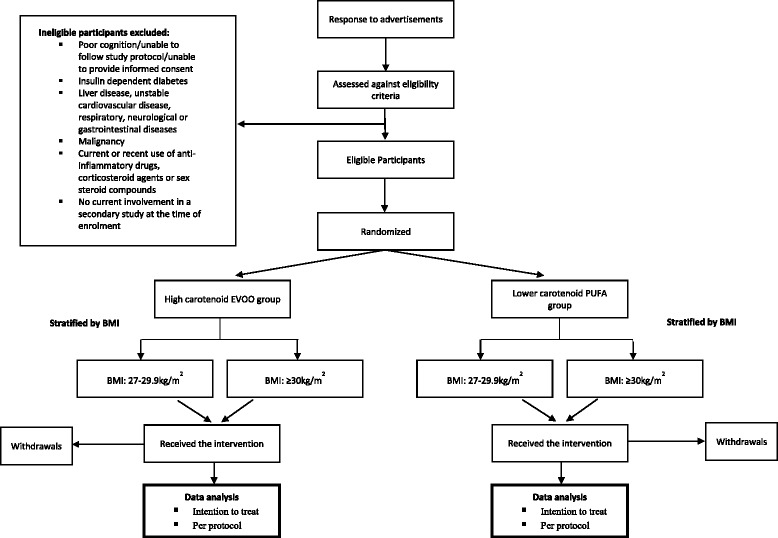
Flow diagram for study participants

Eligible participants will be provided with detailed study information and consent form via post or email, and scheduled to attend a baseline appointment upon providing study consent. At the baseline appointment, body mass, height and BMI will be measured to confirm eligibility. Participants will be recruited via rolling recruitment and randomly allocated to a 12-week prescribed and individualized energy restricted diet enriched with high carotenoid fruits and vegetables and EVOO; or, a 12-week prescribed and individualized energy restricted diet enriched with lower carotenoid fruits and vegetables and a polyunsaturated fatty acid (PUFA) based oil. Volunteers will be randomly assigned to either group following the completion of all baseline measurements. Allocation will be stratified by BMI (27–29.9 kg/m^2^; ≥30 kg/m^2^) using a random number, computer generated randomisation schedule generated and managed by a member of the research team. An overview of the recruitment method and randomisation process is provided in Fig. 1.

All participants will attend the weight management clinic at the University of the Sunshine Coast at baseline and every fortnight for the duration of the study. This study will be conducted according to the guidelines laid down in the Declaration of Helsinki and all procedures involving human participants have been approved by the Human Research Ethics Committee (A/16/801), University of the Sunshine Coast, Queensland, Australia. This trial has been registered with the Australian and New Zealand Clinical Trials Registry (reg no: ACTRN12616001400459). Written informed consent will be obtained from all participants before commencement.

### Data collection

The timeframe for this study will be 18 months, from 1 July 2016 to 30 April 2018. This timeframe includes the collection of all outcome measures and statistical analysis pertaining to this study**.** Assessment of all outcome measures (primary or secondary) will be conducted at baseline and Week 12. The primary outcome measure for this study will be ASM. Secondary outcomes will include body weight, FFM, FM, muscle strength, physical performance, physical activity and health related quality of life. Participants will also report to the clinic each fortnight for the duration of the study where they will be weighed and receive individual dietary counselling by an Accredited Practising Dietitian (APD).

In preparation for all outcome assessments, participants will present to the clinic after an overnight fast wearing light figure hugging clothing without metal zips, tags, or studs. Following the completion of all baseline assessments, participants will be provided with a 3-day food diary and commence a one-week ‘run-in’ period before beginning the 12-week intervention. During the ‘run-in’ week, participants will be instructed to consume their habitual dietary intake. Throughout this period, participants will be asked to record all food and fluid intake across three days (two consecutive weekdays and one weekend day). Upon completion of the ‘run-in’ week, participants will return to the weight management clinic (Week 0) where food diaries will be verbally cross-checked for completeness by an APD, and any ambiguities will be clarified. Participants will be re-weighed following completion of the ‘run-in’ week.

### Anthropometry and body composition

Body mass will be recorded to the nearest 0.1 kg using a calibrated digital scale (AND Weighing; HW-KGL, Melbourne, Australia) with participants wearing light figure hugging clothing and without footwear. Height will be measured to the nearest 0.1 cm whilst barefoot using a wall-mounted stadiometer (Holtain Limited, Crymych, United Kingdom), with the participant’s head positioned in the Frankfort plane. BMI will be calculated as weight (kg) divided by the square of height (m^2^).

Whole-body and regional body composition will be estimated using Dual-energy X-ray absorptiometry (DXA) (Lunar iDXA; GE Healthcare, Madison, WI) with analysis performed using the GE enCORE bone densitometry software (version 16; GE Healthcare). The system software provides estimates of FFM, lean soft tissue, FM and bone mineral density (BMD) for total body and body segments including both arms, both legs and the trunk. ASM was calculated as the sum of lean soft tissue mass in both arms and legs [55], with the ASM index calculated using the formula, ASM/height^2^ (kg/m^2^) [56]. Prior to each scan, participants will be overnight-fasted and not undertaken any vigorous physical activity on the morning of the scan. Scans will be performed with participants wearing light figure hugging clothing without metal zips, tags, or studs and all jewellery and other metal objects will be removed before each scan. Participants will be asked to void their bladder before each scan. All scans will be performed and analysed by the same trained technician.

Quality-assurance and quality-control measures will be performed three times per week throughout the duration of the trial and before all participant scans using a body composition phantom block containing a known bone mineral density and bone mineral content value. Tolerance for the densitometer will be established from the mean of the phantom BMD by monitoring the densitometer’s performance, using ≤1% as the acceptable tolerable limit. All DXA scans will be performed according to the Nana et al. protocol [57] which involves participants lying centrally in the scanning area with their head positioned in the Frankfort plane and with their feet placed in custom made foam blocks to maintain a constant distance of 15 cm between the feet for each scan. Similarly, participants’ hands will be placed in custom made foam blocks so that they will be in a mid-prone position with a standardized gap (3 cm) between the palms and trunk [57, 58]. These custom made blocks are made of Styrofoam and are transparent under DXA. In addition, two Velcro straps will be used to minimize movement during the scan and provide a consistent body position for subsequent scans. One strap will be secured around the ankles and the other strap around the trunk at the level of the mid forearms. The Nana et al. [57] positioning protocol has previously been shown to enhance measurement precision, and minimize the typical errors associated with technical and biological variability of DXA measurements of whole and regional body composition when compared against the traditional NHANES protocol [57, 58].

### Isometric hand-grip strength

Isometric hand-grip strength will be used as a reliable and valid surrogate measure of overall muscular strength [59, 60]. Grip-strength will be measured on both hands with a calibrated hand dynamometer (Smedley, Tokyo, Japan). All measures will be performed with the participant seated in an upright position, with the arm of the measured hand unsupported parallel to the body. Before each measure, the width of the dynamometer’s handle will be adjusted to each participant’s hand size so that the middle phalanges rest on the inner handle. Participants will then be instructed to exert maximal force. For each participant, one ‘test’ trial will be allowed before three consecutive test measurements are undertaken, alternating between each hand, with ~60-s rest intervals between each measure. The mean of the three measures from each hand will be used for the purpose of analyses. All measures will be recorded to the nearest 0.5 kg.

### Physical performance battery

The Short Physical Performance Battery (SPPB) is a well-established, reliable and valid measure of lower extremity performance [61, 62]; the SPPB evaluates lower extremity function by measuring three domains of physical function which mimic activities of daily living: 1) balance; 2) usual gait speed; 3) chair stands. For the assessment of balance, participants will be required to maintain 3 hierarchical standing postures (feet together, semi-tandem, full tandem) for up to 10 s. Usual gait speed will be assessed over a measured distance of 4-m (4-m) where participants will be asked to walk the measured distance at their usual walking speed, without acceleration. Participants will be asked to complete the 4-m walk on two separate occasions. The total time of each measure will be recorded using a stopwatch, with the mean of the two measures used for the purpose of analyses. For chair stands, participants will be instructed to perform a timed test of five repetitions of rising from a seated position and subsequently returning to their original seated position as quickly as possible, without upper extremity assistance (i.e. arms folded across their chest). The time taken to complete the 5 sit to stands will be recorded. This measure will be performed in duplicate with the mean of the two measures used for the purpose of analyses. For consistency, the same chair will be used for all assessments. Results from each of the 3 tests will be ranked using a 0–4 scale, with a value of 0 indicating inability to complete the test and 4 being the highest level of performance [63]. The composite score will be summed, with scores ranging from 0 (worst performance) to 12 (highest level of performance) [63].

### Physical activity

Physical activity in the previous week will be assessed using the long version of the self-administered International Physical Activity Questionnaire (IPAQ-L). Although originally developed for application in adults between 18 and 65 years, previous studies have supported use of the IPAQ-L for estimates of physical activity in older adults ≥65 years [64, 65]. The IPAQ-L assesses duration, frequency and intensity of activities across a range of domains including recreation/leisure, work, transportation and household/yard. Data obtained from the IPAQ-L will be used to estimate the total amount of physical activity completed in a 7-day period by weighting the reported minutes per week in each domain by a metabolic equivalent (MET) energy expenditure estimate. MET minutes per week will then be calculated by multiplying the duration (minutes), frequency (days) and MET intensity, and summing across the different domains, which include vigorous, moderate and walking, to produce an estimate of total physical activity per week (MET-min/wk.^−1^) [66]. Data on total sitting time will also be collected from the IPAQ-L with participants asked to report time spent sitting while at work, at home and leisure-time during the previous 7 days.

### Health related quality of life

Quality of life will be assessed using the self-administered short-form 36 (SF-36) health survey which covers health related quality of life across mental and physical domains. The SF-36 health survey is a 36-item questionnaire assessing eight domains of health and quality of life: physical functioning, role limitations due to physical health, pain, general health, vitality, social functioning, role limitations due to emotional problems and emotional wellbeing. Scoring of the questionnaire will be assembled using the Likert scaling method for summated ratings with raw scores transformed into 0–100 scales, with 0 and 100 assigned to the lowest and highest possible value, respectively. Higher transformed scores indicate a better perceived health status [67]. For the purpose of the present study, the Australian adaptation [68] will be used.

### Baseline questionnaires

Participants will be asked to complete an additional two questionnaires at baseline only; 1) demographic questionnaire: participants will be asked to identify age, gender, level of mobility, use (if any) of community services at home, medication and supplement use; 2) 14-item Mediterranean Diet adherence questionnaire: At baseline, an APD will complete the 14-item Mediterranean Diet adherence questionnaire in a face-to-face interview with each participant. This brief, 14-item questionnaire has previously been validated against a full-length 137-item FFQ [69] for the assessment of adherence to a Mediterranean-style diet. Each item in the questionnaire is scored as 0 or 1, yielding a maximum score of 14 (greatest adherence) [70]. Data from this questionnaire will be used to identify associations between adherence to a Mediterranean Diet and risk of sarcopenic symptomology in future cross-sectional analyses.

### Dietary protocol

All participants, independent of their group allocation, will be prescribed an energy restricted, isocaloric diet with the macronutrient distribution being 30% protein (≥1.2 g/kg), 30% carbohydrate and 40% fat. Caloric restriction will be determined from calculations of individual estimated energy requirements using gender specific predictive equations derived by Mifflin et al. [71]. Individual requirements will be calculated and then reduced by ~30% (2100–4200 kJ/day) to facilitate ~0.5–1.0 kg weight loss per week; as the intervention progresses, the prescribed caloric restriction will be adjusted in order to achieve the desired rate and amount of weight loss. Diets will be presented in the form of a checklist and established using three different energy levels (5000–6000 kJ/d; 6000–7000 kJ/d; 7000–8000 kJ/d).

As a component of the energy restricted diet, participants will be randomised to receive either: a) 375 g/d of high carotenoid vegetables, 300 g/d high carotenoid fruit, and 40-60 ml EVOO; or b) 375 g/d of lower carotenoid vegetables, 300 g/d lower carotenoid fruit, and 40-60 ml PUFA based oil. The serving size of EVOO and PUFA oil will be dependent on the prescribed energy level of the participant. Due to the versatility of EVOO, participants randomized to receive the PUFA based oil will be counselled on using any number of PUFA based oils of choice, including sunflower, rice bran, canola or peanut oils, however participants will be instructed not to consume EVOO. A list of the key prescribed fruits and vegetables (high or lower carotenoid) is presented in Table 2.

**Table 2:** High versus lower carotenoid fruits and vegetables^a^

High Carotenoid Fruits and Vegetables^b^	Lower Carotenoid Fruits and Vegetables^c^
Fruit	Fruit
Rockmelon, watermelon, strawberries, raspberries, mango, blueberries, blackberries, red grapes, oranges, plums, pineapple, papaya, peach, nectarine, apricot, grapefruit, Avocado	Apples, pears, bananas, kiwifruit, green grapes, mandarin
Vegetables	Vegetables
Sweet potato, pumpkin, carrot, red capsicum, yellow capsicum, broccoli, kale, tomato, spinach, dark green lettuce, corn, red cabbage, beetroot, eggplant	White potato, cauliflower, green capsicum, mushroom, iceberg lettuce, peas, green beans, green cabbage, fennel, parsnip, cucumber

*Abbreviations: EVOO* Extra-virgin olive oil, *PUFA* Polyunsaturated fatty acids

^a^Depending on their allocation, participants will be asked to consume fruits and vegetables from the allocated list only

^b^Participants randomized to the high carotenoid plus EVOO group will be asked to consume 375 g/d of high carotenoid vegetables and 300 g/d high carotenoid fruit

^c^Participants randomised to the lower carotenoid plus PUFA oil group will be asked to consume 375 g/d of lower carotenoid vegetables and 300 g/d lower carotenoid fruit

All participants will receive individual dietary counselling by an APD and will be provided detailed instructions on how to maintain and record their daily dietary checklist. If any deviations are made from the prescribed quantities in the checklist, participants will be instructed to record this. All participants will be provided with a selection of recipes, reflecting the composition of the dietary protocol. In addition, all participants will be provided a list outlining the type and variety of fruits and vegetables required for consumption. If participants request mid-meal snacks between meals, they will be counselled on selecting high protein based options. Volunteers will return the checklists every fortnight and these will be discussed with the APD to clarify serving sizes and address any potential issues or problems with compliance toward the dietary protocol. As an additional measure of adherence to the dietary protocol, body weight will be recorded each fortnight for the duration of the study. All participants will be asked to maintain their habitual level of physical activity for the duration of the study. All individual consultations will last between 20 and 30 min in duration. To facilitate compliance, all participants will be provided with a sample fruit and vegetable hamper valued at $30AUD each fortnight for the duration of the study, reflecting the key fruits and vegetables specific to their group allocation. Participants randomized to the high-carotenoid fruits and vegetables, plus EVOO group will also receive EVOO for the duration of the study. All other food(s) prescribed in the energy restricted diet will be provided by the participant.

### Power calculations, intention to treat and statistical analyses

This study was powered on the primary outcome of change in ASM. Based on data from a previous intervention assessing preservation of ASM during intentional weight loss in obese older adults [22] it was estimated that *n* = 56 participants would provide 80% power to detect a significant (*P* < 0.05, 2-sided) change in ASM between the two study groups of 0.9 kg ± 1.2 kg. Assuming an attrition rate of 30%, the target sample size will be *n* = 73 participants. All analyses in the proposed study will be performed using intention-to-treat analysis, with all randomised participants included in the final analysis irrespective of compliance.

Non–normally distributed variables will be logarithmically transformed before analysis. Where normality is not achieved, non-parametric methods for analyses will be used. Descriptive statistics will be presented for baseline characteristics, and independent sample t-tests or chi square analysis will be used to ensure that there are no significant differences between randomised groups. Paired t-tests will be used to identify whether outcome parameters change over time. After screening for assumptions related to multicollinearity, normality, linearity, homogeneity and reliability of covariates, analysis of covariance (ANCOVA) will be applied to identify differences between groups over time, with diet groups as the independent variable and outcome parameters as the dependent variable. Covariates will be determined using a correlation matrix to identify significant interactions between potential confounders. Analyses will be performed using SPSS for Windows 24.0 software (SPSS Inc., Chicago, IL, USA) with statistical significance set at *P* < 0.05.

## Discussion

This study presents a novel intervention examining the efficacy of a high protein diet rich in carotenoids and polyphenols on attenuation of ASM during dietary energy-restricted weight loss in overweight and obese older adults.

To the best of our knowledge, this study will be the first of its kind to examine the efficacy of carotenoid rich fruits and vegetables plus EVOO on attenuating the loss of ASM during energy intake restriction. Traditionally, aggressive weight loss interventions have not been advocated for obese older adults, particularly without the addition of resistance training, due to the potential exacerbation of sarcopenic symptomology and functional decline. Reflecting these concerns, dietary strategies examining the efficacy of energy restricted diets without the addition of structured physical activity is poorly investigated. Notably, many obese older adults have multiple co-morbidities and/or physical limitations that limit their ability to engage in resistance training or other modes of physical activity. It has been suggested that increasing the protein content of the diet may be an effective dietary strategy to attenuate the loss of lean tissue during energy intake restriction in obese older adults [72]. However, several intervention studies have failed to confirm this hypothesis [21, 25]. In contrast, Verreijen et al. [22] reported that a whey protein supplement, enriched with leucine and Vitamin D combined with resistance training in obese older adults preserved ASM during energy intake restriction. Similar results were found by Amamou et al. [26] using an energy restricted high protein diet with and without resistance training. These findings, in part, could relate to the resistance exercise component of the intervention, which is a well-known intervention that stimulates MPS and promotes muscle hypertrophy in older adults when performed progressively over time [73]. Nevertheless, the combination of high-quality protein ingestion coupled with resistance training during energy intake restriction is likely to enhance MPS and attenuate the loss of skeletal muscle. The discrepancy in the literature amongst geriatric obesity weight loss trials without a prescriptive exercise intervention may be attributed to differences in the selected study population (i.e. community dwelling versus frail and/or institutionalized obese older adults), adherence to the dietary intervention and the application of the protein intervention (i.e. total prescription of daily protein intake relative to body weight and/or distribution of protein across the day). Consistent with current recommendations [20, 32], protein will be prescribed ≥1.2 g/kg body weight. Moreover, to account for the potential blunted post prandial response to the anabolic stimuli from dietary protein or amino acids that is associated with ageing, protein at each meal will be enhanced to include at least 20-30 g of high-quality protein per meal [27]. Whether per meal enhancement of protein intake can offset a lack of physical activity to preserve ASM and function during energy intake restriction in overweight and obese older adults is unknown.

A strength of the proposed study is a consideration for the synergistic relationship of nutrients and their potential influence on skeletal muscle function and body composition. Although the pathophysiology related to sarcopenic symptomology is multifactorial and complex, excessive oxidative stress caused by the accumulation of free radicals and low-grade systemic inflammation have been recognized as key contributors toward age-related declines in SMM and function [74, 75]. The proposed health benefits of carotenoids and polyphenols, including their impact on healthy ageing have been extensively reviewed [76–79]. Importantly they are known to be efficient quenchers of singlet oxygen, as well as potent scavengers of other reactive oxygen species (ROS), inhibit lipid peroxidation and modulate redox-sensitive transcription factors involved in the up-regulation of pro-inflammatory cytokines [76, 78, 80]. Given previous epidemiological evidence showing that carotenoids and polyphenols are inversely associated with sarcopenic symptomology, it is biologically plausible that these nutrients may preserve skeletal muscle and function during energy intake restriction in overweight and obese older adults. Intervention studies however are scant.

A limitation of the present study is a lack of resources available to collect blood or urinary samples for the analyses of plasma carotenoids and/or urinary excretion of polyphenols as a measure of compliance to the dietary protocol. Furthermore, given that fruits and vegetables contain a wide range of nutrients and bioactive compounds, elucidating exactly which component is responsible for any potential observed benefit relating to preservation of lean body mass, muscle strength and physical performance parameters remains challenging. Therefore, the biological plausibility to support a causal role of fruit and vegetable intake, or indeed dietary carotenoids as a dietary strategy to attenuate sarcopenic symptomology must take into account the synergistic relationship of nutrients within fruits and vegetables. Furthermore, using a self-reported physical activity instrument to estimate physical activity has potential to misreport physical activity levels relative to the use of accelerometry monitors for estimates of physical activity, sedentary behaviour, sleep and total daily energy expenditure.

Currently, weight loss diets for overweight and obese older adults remain heavily debated due to the potential risk for the loss of skeletal muscle and threat to functional decline. Based on this argument, weight loss interventions for older overweight and obese adults must focus on the preservation of skeletal muscle and function. Future publications will detail major outcomes from this proposed study whilst providing a better understanding related to the influence of diet induced weight loss on skeletal muscle and its impact on muscle strength and physical performance parameters in overweight and obese older adults.

## Abbreviations

APD: Accredited practising dietitian
BMD: Bone mineral density
BMI: Body mass index
DXA: Dual energy X-ray absorptiometry
EVOO: Extra virgin olive oil
FFM: Fat free mass
FM: Fat mass
IPAQ-L: International physical activity questionnaire
MPS: Muscle protein synthesis
PUFA: Polyunsaturated fatty acid
RCT: Randomized control trial
SF-36: Short-form 36 health survey
SMM: Skeletal muscle mass
SPPB: Short physical performance battery
